# A Review of the Design of Multimedia Patient Educational Materials in Low Back Pain Research

**DOI:** 10.1298/ptr.R0032

**Published:** 2024-06-28

**Authors:** Garett VAN OIRSCHOT, Cailbhe DOHERTY

**Affiliations:** ^1^School of Public Health, Physiotherapy & Sport Science, University College Dublin, Ireland; ^2^Insight SFI Research Centre for Data Analytics, Ireland

**Keywords:** Health education, Patient education, Patient education materials, Multimedia, Low back pain, E-health, Self-management

## Abstract

Low back pain guidelines recommend patient education as a component of management. Multimedia education materials to provide patient education are increasingly being used not only due to the convenience of digital services but also because this is an efficient way to deliver educational information to under-resourced or rural/remote regions without optimal healthcare services. To maximize the knowledge transfer of research findings and low back pain guidelines, scientifically backed information must evolve beyond journal prints, bland government websites, and the basic web design of budget-constrained advocacy groups. Materials must instead be engaging for the public and compete with the various sources of low back pain misinformation, which can appear attractive and eye-catching while being conveniently accessed. We discuss a data subset from a larger musculoskeletal healthcare review to highlight the educational materials used in low back pain randomized controlled trials found in the literature. While there is no standard way to appraise the effectiveness of such educational materials, potential options are discussed. Future research is needed to determine whether knowledge is being transferred and whether this is the avenue to improving patient outcomes.

## Introduction

Low back pain (LBP) has a significant burden on society, with worldwide prevalence reported as high as 84% and 11%–12%, with those with the condition considered disabled^1)^. It continues “to be the greatest cause of disability burden worldwide, and two-fifths of this burden has been attributed to modifiable risk factors”^2)^. Persistent cases are expected to have continued high levels of pain and disability^3)^.

Mirroring best practice for most musculoskeletal healthcare^4)^, the recommended management for LBP across many international guidelines includes patient education^5)^. Patient education aspires to empower patients to participate in and adhere to their treatment^4^^,^^6^^–^^8)^. Especially in an era when health systems must cope with chronic musculoskeletal (MSK) conditions, treatment approaches must pivot from curing to self-management^9)^. While the content of any patient education materials (PEMs) will continue to be presented and discussed in all health-related literature, there is also a worthy discussion to be had surrounding the design characteristics of the educational materials provided to patients when they are given in multimedia formats, defined as any use of images and words^10)^.

Images and words allow us to provide information that may be processed and remembered better than words alone^10–12)^. Educational materials can complement the information relayed in clinical encounters, but in cases of understaffed or rural/remote healthcare systems, they offer an opportunity to relay information to patients when clinical encounters are delayed or not possible^13–15)^. Once established, such materials are then relatively cheap to reproduce and easily distributed over a large, but digitally connected, geographical area^14^^,^^16^^–^^19)^. They can also offer additional features like individual tailoring, remote support, and engagement tracking to allow for increased uptake^20–22)^.

The Cognitive Theory of Multimedia Learning (CTML)^23)^ is an evolving evidence-based framework for optimizing educational content^24^^–^^26)^ that is derived from the pedagogical literature but has also been used in health research. It has informed the design of healthcare education materials provided to practitioners^27–29)^, students^30–34)^, and patients^35–40)^, including patients with MSK-related conditions (hip)^41)^. In trying to maximize the effectiveness of health education, it is incumbent upon researchers, policymakers, and clinicians to ensure the information they provide to patients and the general public is not just accurate but will be attractive and engaging enough to be seen by more people^42)^ who might otherwise be easily swayed by equally attractive and engaging materials that are inaccurate or misinformation^43–45)^. Unfortunately, this competition between scientific advice and misinformation is rife within the MSK healthcare field^46–50)^, of which LBP is a part. Such shortcomings are more likely when research on PEMs lacks sufficient description and reporting standards^13^^,^^51)^.

This review is a secondary analysis of a larger systematic review that examined the educational materials across all musculoskeletal conditions^52)^. It seeks to discuss the LBP subset of studies to better understand what educational materials have been used in LBP research. While the content of LBP education materials has been previously investigated^51)^, this review intends to discuss the types of multimedia used and the design formats preferred. Difficulty has already been noted when attempting to retrieve and examine PEMs used in LBP research^51)^. Another study of LBP videos found a poor correlation between user engagement and location/setting, duration, conflict of interest risk, speaker’s professional designation, source of the video, and clinical recommendations^50)^. This study then recommended future research should examine how audio-visual aspects may affect engagement, illustrating the importance of how reviews of LBP educational materials need to also consider their design and not just content. Theoretically, there may be optimal strategies to implement when designing multimedia PEMs. Such expertise could help to compete with any potential LBP misinformation in existence so examining not just the educational content but also how their design is reported and described could aid in future LBP research.

## Materials and Methods

While described in more detail elsewhere^52^^,^^53)^, a brief description of the methods is as follows: PubMed, CINAHL, PsycINFO, and Embase were searched from inception to 20 September 2023. Inclusion criteria were as follows: Randomized controlled trials of those ≥18 years of age used any multimedia-based education intervention and examined against any comparator. Multimedia education materials included any combination of reusable words and images that were delivered to patients with examples such as infographics, books, pamphlets, and videos. This narrative review is based exclusively on the studies of LBP, defined as pain between the lower edge of the ribs and the buttock^54)^. Exclusion criteria consisted of any interventions that relied on clinician-delivered education with no provision of materials. No date or language restrictions were applied. A secondary reference list scan of included studies was also conducted. Title and abstract screening were conducted via Covidence^55)^, and articles were advanced to full-text review when both authors agreed. All articles in agreement were advanced to the data extraction phase, and any conflicts throughout this process were resolved by consensus between the two authors.

In instances when an included study lacked sufficient detail about the PEMs used, a request for further information was emailed to the study authors. If there was no reply to the initial or follow-up request for study materials, then the study was classified as having interventions that were irretrievable.

Following Mayer’s CTML^10)^ framework, all study interventions were evaluated using its 15 principles, described further in Table 1. In situations where multiple or lengthy PEMs were used in a study, then a sample of the materials was taken from the study with the evaluation of these materials agreed upon between both authors.

**Table 1.** Explanation of Mayer’s Cognitive Theory of Multimedia Learning design principles

Design principle	Explanation
1. Multimedia principle	People learn better from words and pictures than from words alone.
2. Coherence principle	People learn better when extraneous material is excluded rather than included.
3. Signaling principle	People learn better when cues are added that highlight the organization of the essential material.
4. Redundancy principle	People do not learn better when printed text is added to graphics and narration. People learn better from graphics and narration than from graphics, narration, and printed text when the lesson is fast-paced.
5. Spatial contiguity principle	People learn better when corresponding words and pictures are presented near rather than far from each other on the page or screen. For example, in an animation on lightning formation, captions are presented at the bottom of the screen (separated presentation) or are placed next to the event they describe in the animation (integrated presentation).
6. Temporal contiguity principle	People learn better when corresponding words and pictures are presented simultaneously rather than successively. For example, First, the learner views an animation on lightning formation and then hears the corresponding narration or vice versa (successive group), or the learner views an animation and hears the corresponding narration at the same time (simultaneous group).
7. Segmenting principle	People learn better when a multimedia message is presented in user-paced segments rather than as a continuous unit.
8. Pre-training principle	People learn more deeply from a multimedia message when they know the names and characteristics of the main concepts.
9. Modality principle	People learn more deeply from pictures and spoken words than from pictures and printed words.
10. Personalization principle	People learn better from multimedia presentations when words are in a conversational style rather than a formal style. For example, in a narrated animation on how the human lungs work, personalization involves using “you” and “your” in the narration script, such as “your nose” rather than “the nose” and “your throat” rather than “the throat.”
11. Voice principle	People learn better from multimedia presentations when words are spoken in an appealing human voice.
12. Image principle	People do not learn better from multimedia presentations when a static image of the instructor is added to the screen.
13. Embodiment principle	People learn more deeply from multimedia presentations when an onscreen instructor displays high embodiment rather than low embodiment.
14. Immersion principle	People do not necessarily learn better in 3D immersive virtual reality than with a corresponding 2D desktop presentation.
15. Generative activity principle	People learn better when they are guided in carrying out generative learning activities during learning (e.g., summarizing, mapping, drawing, imagining, self-testing, self-explaining, teaching, or enacting). For example, after each of the six sections in a virtual reality simulation of the human bloodstream, students are asked to verbally summarize what they have learned.

## Results

The entire data can be found on Open Science Framework via https://osf.io/6j59s/?view_only=8d3cd39f67d1489f992f0ecb5e846684.

This review discusses 41 studies in which multimedia educational materials were provided to 12,011 participants with LBP. All 41 studies were conducted between 1995 and 2022, with over half (54%) conducted from 2018 onwards. Female names accounted for 15 (36%) of primary authors. Further information on these studies and on the participants is found in Tables 2 and 3.

**Table 2.** Summary of study characteristics

Country	N	%
EU^a^ (countries shaded below)	17	41.5%
USA	5	12.2%
UK	5	12.2%
Spain	4	9.8%
Germany	3	7.3%
Australia	3	7.3%
Brazil^*^	3	7.3%
Iran^**^	3	7.3%
France	2	4.9%
Saudi Arabia	2	4.9%
Denmark	1	2.4%
Sweden	1	2.4%
Ireland	1	2.4%
China^*^	1	2.4%
Finland	1	2.4%
Korea	1	2.4%
Thailand^*^	1	2.4%
Jordan^*^	1	2.4%
Turkey^*^	1	2.4%
Nigeria^**^	1	2.4%
Croatia	1	2.4%
Total	41	100.0%
High income	30	73.2%
^*^Upper middle income	7	17.1%
^**^Lower middle income	4	9.8%

^a^European Union

^*^Upper middle-income rating as per World Bank

^**^Lower middle-income rating as per World Bank

**Table 3.** Summary of patient characteristics

	N	%
Gender		
Male	6324	52.7%
Female	5376	44.8%
Not reported	311	2.6%
Total	12,011	100%
Age (range^a^)	18–85	
Nationality		
USA	4645	38.7%
EU^b^ (countries shaded below)	4678	38.9%
France	2479	20.6%
UK	1283	10.7%
Germany	886	7.4%
Brazil^*^	638	5.3%
Finland	415	3.5%
Sweden	243	2.0%
Iran^**^	230	1.9%
Denmark	210	1.7%
Spain	209	1.7%
Ireland	206	1.7%
Australia	196	1.6%
Saudi Arabia	105	0.9%
Korea	43	0.4%
Thailand^*^	42	0.3%
Jordan^*^	41	0.3%
China^*^	40	0.3%
Turkey^*^	40	0.3%
Nigeria^**^	30	0.2%
Croatia	30	0.2%
Total	12011	100.0%
High income	10950	91.2%
^*^Upper-middle income	801	6.7%
^**^Lower middle income	260	2.2%

^a^Range was given due to heterogeneous reporting of age

^b^European Union

^*^Upper middle-income rating as per World Bank

^**^Lower middle-income rating as per World Bank

**Fig. 1.**
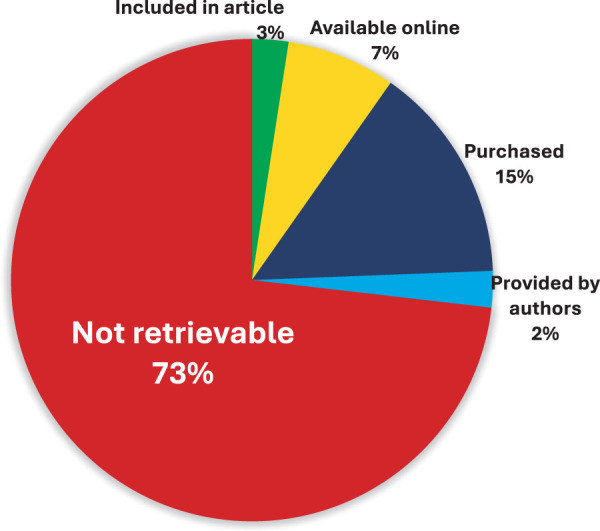
Retrievability of multimedia educational materials in all 41 studies

The types of multimedia interventions used in LBP studies varied, but the most common form was a leaflet that contained images, and the breakdown of all types is shown in Table 3. As detailed in Figure 1, two (3%) of the studies included educational interventions or a way to access the educational interventions within the article, while three (7%) used materials that could be found online. Another six studies used materials that could be purchased and one (2%) study provided their materials when requests were sent to the authors. Note that for materials requiring purchase, this pertained to books (average cost = 11.55EUR per unit). The remaining 30 (73%) of studies contained unretrievable educational materials.

The 11 (27%) studies with retrievable materials were assessed for their conformity to the CTML described earlier, and the results are shown in Table 4. The inter-rater agreement between both authors was 100% after the discussion took place following individual ratings.

**Table 4.** Conformity to Cognitive Theory of Multimedia Learning for the 11 (27%) studies with retrievable materials

Primary author	Description of educational intervention	Type	1	2	3	4	5	6	7	8	9	10	11	12	13	14	15
Chenot 2019	German version of The Back Book (Rückenbuch)	Book	Yes	No	No	No	Yes	Yes	Yes	No	No	Yes	N/A	N/A	N/A	Yes	No
Coudeyre 2006	The French version of The Back Book (Le Guide du Dos)	Book	Yes	No	No	No	Yes	Yes	Yes	No	No	Yes	N/A	N/A	N/A	Yes	No
Coudeyre 2007	The French version of The Back Book (Le Guide du Dos)	Book	Yes	No	No	No	Yes	Yes	Yes	No	No	Yes	N/A	N/A	N/A	Yes	No
Gardner 2019	Participant Handbook	Manual/Workbook	Yes	No	No	No	Yes	Yes	No	Yes	No	Yes	N/A	N/A	N/A	Yes	Yes
George 2009	The Back Book	Book	Yes	No	No	No	Yes	Yes	Yes	No	No	Yes	N/A	N/A	N/A	Yes	No
Gibbs 2022	Pain education TEDx video: https://www.youtube.com/watch?v=gwd-wLdIHjs	Video (or film)	Yes	Yes	No	Yes	N/A	Yes	No	Yes	Yes	Yes	Yes	Yes	Yes	Yes	No
Lamb 2010	The Back Book	Book	Yes	No	No	No	Yes	Yes	Yes	No	No	Yes	N/A	N/A	N/A	Yes	No
O’Keeffe 2020	CFT written info	Leaflet/Pamphlet/Booklet	Yes	No	Yes	No	Yes	Yes	Yes	Yes	No	Yes	N/A	N/A	N/A	Yes	Yes
Saper 2017	DVD and home practice manual	Multiple	Yes	No	Yes	No	Yes	Yes	Yes	No	No	Yes	N/A	N/A	N/A	Yes	No
Sherman 2005	Yoga & exercise leaflets	Leaflet/Pamphlet/Booklet	Yes	No	Yes	No	Yes	Yes	Yes	No	No	Yes	N/A	N/A	N/A	Yes	Yes
Simula 2021	Booklet (www.mq.edu.au/about/about-the-university/our-faculties/medicine-and-health-sciences/departments-and-centres/department-of-health-professions/our-research/low-back-pain-management-booklet)	Leaflet/Pamphlet/Booklet	Yes	No	Yes	No	Yes	Yes	No	Yes	No	Yes	N/A	No	No	No	No

N/A = not applicable due to the nature of educational materials or due to the inability to translate the language of materials

Links are included where materials were found freely online.

These 11 studies were appraised according to the CTML principles (Table 4). Where the multimedia contained audio, 100% of interventions abided by the voice principle using an appealing human voice and the personalization principle of addressing the listener personally as “you” in a conversational style. All interventions conformed to the principle of spatial contiguity by displaying text and graphics in close proximity, as well as temporal contiguity by presenting text and graphics simultaneously. Ten (91%) studies abided by the principle of immersion by avoiding virtual reality, and eight (73%) studies conformed to the segmenting principle by presenting educational material in shorter segments instead of continuously.

This was less conformity to the remaining CTML principles. Half of the studies showed adherence to the image principle using moving images of speakers on screen and the embodiment principle of displaying the speaker instead of having them “off-screen.” Four (31%) studies applied the signaling principle, where cues are used to organize the information, as well as the pre-training principle by familiarizing participants with the main concepts in advance. Three (27%) studies applied the principle of generative activity by including a generative learning activity for the learner.

The principles with the least presence were the principles of modality, where pictures are accompanied by spoken words over written words, coherence when excessive or extraneous information is excluded, and redundancy where redundant text alongside graphics is avoided. These were each only present in one study.

### Outcome measures

Of the 41 included studies, one study reported on the primary outcome for this review, knowledge.

For the secondary outcome of any patient-reported measures, pain intensity was the most frequent and included visual analog or numerical ratings used in 28 (68%) studies. The next most common outcome measure was the Roland Morris Disability Questionnaire, used in 18 (43%) studies, followed by the Oswestry Disability Index in 15 (36%) studies, and the Fear Avoidance Beliefs Questionnaire, used in 10 (24%) studies.

## Discussion

The authors express concern that multimedia PEMs could only be retrieved for 11 (27%) studies, and just 4 (10%) were available with the article or freely online. Conducting a review of PEM intervention used in LBP research is extremely hampered when the interventions cannot be examined. Nearly three-quarters of studies could not have their interventions appraised. This will additionally prevent replication studies from being conducted. While educational materials are often intertwined with intellectual property considerations, it should be pointed out that much of this type of academic research is publicly funded to inform clinical practice. A conflict of interest arises when public support is used to create interventions that cannot be shared, examined, or replicated for public benefit. Patient education is meant to be one pillar of management in a variety of guidelines in MSK healthcare^8^^,^^56^^–^^64)^ and naming or describing the intervention in place of providing it does not help clinical practice.

If the reporting issues in patient education research for low back occurred in other areas like exercise prescription^65^^,^^66)^ and pharmacologic mangement^67)^, it would be justly criticized. The authors contend that patient education research must be held to the same standard. Open science principles should continue to be encouraged and researchers should heed these principles through the use of online repositories and persistent identifiers.

Patient education interventions for LBP also lack a standardized method of appraising their design and delivery. The authors wanted to evaluate the studies in this review using a framework focused on design and learning, and the CTML was the best option to be found that does not preclude a better method from being developed. It has been used in a body of healthcare research already^50^^–^^63)^ with an appearance in musculoskeletal healthcare^41)^ but not necessarily in the LBP domain. If a greater sample of interventions could be obtained and evaluated, it would be informative to examine if a larger sample lacked the similar CTML principles found in our small sample of 11 studies that provided their interventions. It would be even more informative to examine if implementing all of the CTML principles led to improved outcomes. What can be said is that if the CTML provided a way to ensure better learning for patients as found in its pedagogical historical foundations^10)^, healthcare providers could provide more engaging and educational materials to patients. While advice exists on improving content^68)^, delivery^69)^, and understandability^26)^ in healthcare, the CTML might also show how to improve the design quality of materials to maximize engagement and learning. In this relatively small sample where the educational materials of 11 studies were obtained, the same four principles of coherence, redundancy, and modality are commonly overlooked. These principles are relatively easy to adapt in the text and graphic design of websites, apps, or social media posts that are disseminated to patients and the general public. There is already advice in the musculoskeletal literature about segmenting into shorter portions^70)^ or personalizing the narration/experience^71)^ in line with CTML principles^10)^.

One might question why such scientific advice on LBP needs to provide education that is engaging. The authors would point to the misinformation that exists in healthcare^43^,^44)^ and that LBP education does not necessarily win this contest through debate or debunking alone^72)^ but also needs to be presented with appeal and attraction in this current era of rapidly produced social media content^71)^ where healthcare information must maximize engagement with multimedia such as videos^70^^,^^73)^.

The authors acknowledge that knowledge improvement may not be the key ingredient in advancing clinical outcomes, but we contend that it has not been examined comprehensively in the PEM-related research on LBP. We noted that just one out of 41 studies tested knowledge as an outcome, despite all 41 studies using educational material as an intervention. This suggests that knowledge was rarely the outcome of interest in these studies, despite the aforementioned guidelines recommending patient education as part of LBP management^5)^. The relationship between patient education and improved clinical outcomes for LBP should be supported by some sort of investigation into their association. It has already been noted that knowledge is under-assessed in LBP research^51)^, and our review similarly found that disability, function, and pain were more heavily favored in the studies included here.

One limitation of reviewing the literature for this review was the lack of representation. jurisdictions with under-resourced or very remote healthcare systems are not well represented when examining the upper and lower-middle-income countries from Table 2. These are the precise situations where healthcare services may have a keen interest in the design of multimedia PEMs so that they can be leveraged when one-to-one clinical care is impeded by resources or geography.

Perhaps healthcare providers of LBP are ill-equipped to advance their expertise in designing engaging education materials for multimedia consumption. Smaller organizations may never be able to train such expertise with in-house staff. Perhaps it is worth considering the benefits of liaising with social media content creators to exploit this skill set.

Finally, the authors would strongly support more rigorous reporting standards when it comes to patient education research into LBP. It should be specified clearly whether the materials are used in the form of a graphic, video, leaflet, etc., and an indication of length like word count, time, or number of pages. While being described in detail is helpful, this should be in addition to the open science principles of providing the PEM interventions as an online appendix or supplemental information or within an online repository. Also, if education is being used as an intervention, then some evaluation of knowledge should be employed.

## Conclusion

Multimedia PEMs are widely used in LBP research healthcare, yet this review outlines how they are frequently not supplied or sufficiently described to achieve adequate appraisability or reproducibility. Patient education should strive for better reporting standards so that its implementation in LBP care can be better replicated. While no studies in our small sample appeared to optimize the design of their multimedia PEMs in accordance with the CTML framework, this could be addressed by encouraging more design reconfigurations that incorporate generative learning activities, use of audio over text where possible, avoiding extraneous details, and avoidance of redundant text alongside graphical information. Knowledge transfer and retention must be better assessed to explore the mechanisms of patient education.

## Funding

This research did not receive any specific funding.

## Availability of Data and Materials

Data and materials are available at OSF at https://osf.io/6j59s/?view_only=8d3cd39f67d1489f992f0ecb5e846684.

## Conflicts of Interest

The authors have no conflicts of interest to declare.
